# FCoV Viral Sequences of Systemically Infected Healthy Cats Lack Gene Mutations Previously Linked to the Development of FIP

**DOI:** 10.3390/pathogens9080603

**Published:** 2020-07-24

**Authors:** Mirjam Lutz, Aline R. Steiner, Valentino Cattori, Regina Hofmann-Lehmann, Hans Lutz, Anja Kipar, Marina L. Meli

**Affiliations:** 1 Clinical Laboratory, Department of Clinical Diagnostics and Services and Center for Clinical Studies, Vetsuisse Faculty, University of Zurich, CH 8057 Zurich, Switzerland; lutz@immunology.uzh.ch (M.L.); aline_steiner@gmx.ch (A.R.S.); valentino.cattori@gmail.com (V.C.); rhofmann@vetclinics.uzh.ch (R.H.-L.); hanslutz@me.com (H.L.); 2 Institute of Veterinary Pathology, Vetsuisse Faculty, University of Zurich, CH 8057 Zurich, Switzerland; anja.kipar@uzh.ch

**Keywords:** healthy FCoV carriers, FIP, mutations, S gene, ORF3, ORF7, experimental infection

## Abstract

Feline Infectious Peritonitis (FIP)—the deadliest infectious disease of young cats in shelters or catteries—is induced by highly virulent feline coronaviruses (FCoVs) emerging in infected hosts after mutations of less virulent FCoVs. Previous studies have shown that some mutations in the open reading frames (ORF) 3c and 7b and the spike (S) gene have implications for the development of FIP, but mainly indirectly, likely also due to their association with systemic spread. The aim of the present study was to determine whether FCoV detected in organs of experimentally FCoV infected healthy cats carry some of these mutations. Viral RNA isolated from different tissues of seven asymptomatic cats infected with the field strains FCoV Zu1 or FCoV Zu3 was sequenced. Deletions in the 3c gene and mutations in the 7b and S genes that have been shown to have implications for the development of FIP were not detected, suggesting that these are not essential for systemic viral dissemination. However, deletions and single nucleotide polymorphisms leading to truncations were detected in all nonstructural proteins. These were found across all analyzed ORFs, but with significantly higher frequency in ORF 7b than ORF 3a. Additionally, a previously unknown homologous recombination site was detected in FCoV Zu1.

## 1. Introduction

Feline Coronaviruses (FCoVs) are endemic in the domestic cat population and are found worldwide with high seroprevalences. FCoVs are positive-stranded enveloped RNA viruses with a non-segmented genome of ~29 kilobases (kb) encoding for a polymerase (replicase complex 1a and 1b), four structural proteins (spike (S), membrane/matrix (M), envelope (E), and nucleocapsid (N)), and five nonstructural/accessory proteins (NSP) 3abc and 7ab [1]. Due to infidelity of the RNA polymerase, FCoVs show high mutation rates upon replication. This leads to the formation of quasispecies, a cloud-like appearance of multiple genetic virus variants that are linked by mutations [2,3]. Virus variants that acquire high virulence can lead to Feline Infectious Peritonitis (FIP), an immune-mediated disease that is currently the most frequent fatal infectious disease in young pedigree cats and cats in shelters [4,5]. However, the pathogenesis of FIP is still not fully understood [6].

FCoVs occur as two pathotypes, the low virulence so-called feline enteric coronaviruses (FECV) and the virulent FIP viruses (FIPV). The former dominates since FCoV infection is via the fecal–oral route and primarily targets the intestine; it does, if at all, only induce mild enteritis. However, 4–5% of adult cats and 5–10% of kittens develop FIP at some point after infection [7,8] as a consequence of *de novo* occurrence of highly virulent FCoVs that arise from low virulent FCoVs by mutations in the individual infected cat [9]; these are generally not horizontally transmitted. It was initially thought that the essential process for the development of FIP would be a broadening of the viral target cell spectrum from enterocytes to also include monocytes/macrophages, which would be mediated by specific viral mutations and would allow systemic infection, leading to FIP [10]. In vitro studies subsequently revealed that it is effective and sustainable replication in macrophages rather than the capacity to enter the cells that confers virulence of FCoVs [11,12]. Also, it was soon shown that healthy carrier cats become systemically infected and carry low amounts of virus in different organs [13,14].

Irrespective of the pathotype, FCoVs can be classified into two serotypes based on their neutralization reactivity with S protein-specific monoclonal antibodies [15,16]. Serotype II FCoVs have arisen from a double RNA recombination event between canine coronavirus (CCoV) and serotype I FCoVs [17]. Only type II FCoV can be efficiently propagated in cell cultures [5]. However, viruses of both serotypes can cause FIP.

Previous studies found deletions in open reading frame (ORF) 3c and mutations in ORF 7b in FCoVs from organs of cats that succumbed to FIP [9,18]. These were not found in fecal FCoV isolates from healthy carrier cats and were hence considered to be linked to the FIP pathotype and, consequently, to the development of FIP. In fact, an intact ORF 3c was shown to be critical for replication in enterocytes and thus, shedding of the low virulent pathotype FCoVs [19,20]. The fact that the deletions and mutations were unique to each cat supported the *de novo* mutation theory [21]. FCoVs found in FIP often exhibit mutations in ORF 3c that lead to truncation of the protein; however, a significant proportion (up to 40%) of FIPV still have an intact ORF 3c [9,18,20,22,23]. Hence, a virulence switch cannot be explained with ORF 3c deletions alone [18,20]. Nevertheless, mutations that affect the expression of the accessory proteins might contribute to increased viral replication in monocytes/macrophages [24,25] and, thereby, to the development of FIP. Conflicting evidence exists regarding the role of ORF 7a and b. While some studies found deletions in ORF7 to be associated with reduced in vivo virulence or in vitro replication [24,25,26,27], others did not detect such links [28,29,30]. A high N protein diversity was identified as evidence of quasispecies formation, but not as a marker of pathotypes [31]. Mutations in the E or M genes were not found to be critical, neither in vivo [21] nor for in vitro replication [30].

The FCoV S glycoprotein mediates viral cell entry through receptor binding and is thus responsible for cell tropism [32]. Early in vitro studies found that macrophage tropism can be promoted by introducing the S protein of a highly virulent type II FCoV strain into its low virulent counterpart, suggesting that this protein plays a role in virulence. However, the study did not associate a distinct mutation with the new features of the recombinant strain [30]. Specific S gene mutations were only identified in a more recent full genome sequencing approach, analyzing FCoVs of both pathotypes. The majority of FIPV pathotype FCoVs were found to carry a mutation in a nucleotide position that was associated with an amino acid change in the putative fusion peptide of the S protein [33]. The same mutation plus a substitution leading to an amino acid change in the heptad repeat 1 (HR1) region, possibly leading to an altered fusogenic activity of the S protein and thus an altered cellular tropism, was found in another study [34]. However, it was subsequently shown that the amino acid change described by Chang and coworkers [33] correlates with systemic spread of the virus and not directly with FIP [35]. Another study examined a furin cleavage site between the receptor binding and fusion domain of the S gene. The site was found to be conserved in less virulent FCoVs, whereas FIP pathotype FCoVs exhibited a higher variability in the core motif and surrounding residues. These mutations alter cleavability of the S protein by furin. Depending on the exact position and the exchanged nucleotide, cleavage was either enhanced or suppressed [36]. Again, the study design did not consider systemically infected healthy carrier cats in this context. Reverse genetics approaches with recombinant chimeric viruses in which genes of an avirulent (FCoV Black) strain were successively substituted by genes from a highly virulent serotype II FIPV strain (79-1146) led to seroconversion and systemic infection, but did not induce FIP [37,38]. Despite the fact that many comparative studies have described mutations only present in FIP pathotype FCoVs, their relevance for the development of FIP remains unclear.

Based on the hypothesis that certain mutations are essential for the capacity of FCoVs to spread systemically, the present study investigated a cohort of systemically infected healthy carrier cats at different time points post experimental infection for the presence of a range of mutations in the genes encoding for the S protein, NSP 3abc, and NSP 7b, which have been shown to have implications for the development of FIP. It also assessed the changes in the swarm composition of the challenge stock virus present in the organs to determine if its complexity would increase or rather decrease with time.

## 2. Results

### 2.1. Molecular Cloning of Viral Sequences

To track viral sequence mutations in organs of healthy FCoV carrier cats, we investigated FCoV sequences detected in the colon, liver, and thymus, as well as feces of seven experimentally FCoV infected cats. In two animals, the thymus was found to be negative by FCoV reverse transcription quantitative real-time PCR (RT-qPCR) and lung and tonsil were included as alternatives (Table 1). FCoV RT-qPCR was performed from undiluted and 10-fold diluted RNA samples to detect any potential PCR inhibition and the RNA dilutions with the lower cycle threshold (CT) value, corresponding to the higher viral load, were selected for analysis by one-step RT-PCR (Table 1) for further cloning and sequencing.

Due to low tissue viral loads and/or lack of primer hybridization due to possible sequence mismatches, in five of the 28 samples, the analysis of mutations in either ORFs 3abc or 7b was not possible (cat 3257B: amplicon NSP7ab from liver and tonsil; cat D4: amplicon NSP7ab from the liver; cats Y1 and 3138B: amplicons S-E and S-M from the thymus, respectively). In total, 51 sequences were obtained. The plan was to sequence three colonies from each amplicon. Due to the often low concentrations of eluted viral cDNA, this was not possible for all samples. However, sequences for 129 of the expected 168 amplification reactions were obtained.

We determined the sequence of the putative fusion region of the S protein from at least one amplicon from each organ of five cats (3132B, 3138B, 3312B, D4, Y1). The one-step RT-PCR for the S gene already yielded an amplicon of the expected length (615 bp) from the colon of five cats (3132B, 3138B, 3312B, D4, Y1) and the liver of cat 3138B, and after the nested PCR, amplicons with the expected length (134 bp) were obtained from the liver of four cats (3132B, 3312B, Y1, 3257B), the thymus of four cats (3132B, 3138B, 3312B, Y1), and the tonsil of cat 3257B. From one cat (Y2), no amplicon was obtained.

### 2.2. Sequencing of ORFs 3abc and 7b

#### 2.2.1. Swarm Composition and Mutational Frequencies between Challenge Stock and Tissue/Fecal Virus

To determine how a virus swarm changes in its composition and viral sequences during in vivo infection and with systemic spread, the genomic sequences of FCoVs in the tissue and fecal samples were compared to those of challenge stock virus. The number of sequences identical to the consensus was always higher in the latter than in the tissue/fecal counterparts of the same strain and ORF (Table 2).

**Figure 1:**
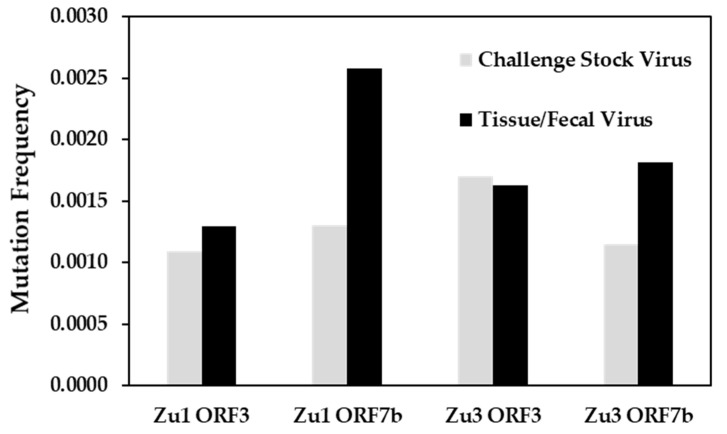
Mean mutation frequencies in challenge stock virus and tissue/fecal viruses for ORF3 and ORF7b of FCoV Zu1 and FCoV Zu3.

The number of single nucleotide polymorphisms (SNPs) per nucleotides analyzed did not differ significantly between challenge stock virus and tissue/fecal viruses. However, a tendency towards higher mutation frequencies was observed for ORF7b in the tissue/fecal viruses compared to the challenge stock virus (Table 2, Figure 1). Interestingly, Zu3 ORF3 showed higher mutation frequencies in the stock virus than in the tissue/fecal viruses.

#### 2.2.2. Comparative Assessment of Mutation Frequencies in Genes 3a, 3b, 3c, and 7b

**Figure 2:**
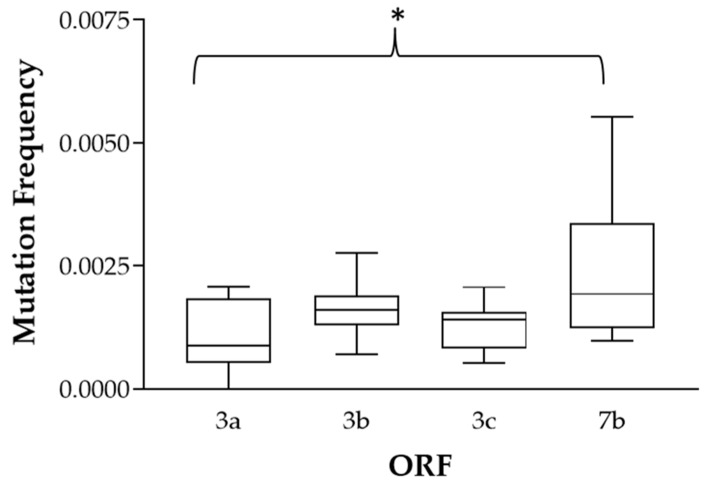
Mutation frequency in the different ORFs. Box plots (boxes showing the 25%, 50%, and 75% quantiles) and whiskers representing minimum and maximum values within each gene; *, *p*-value < 0.05.

To determine whether there was a higher selective pressure on a certain gene after in vivo infection, we compared the mutation frequencies and resulting amino acid changes of genes 3a, 3b, 3c, and 7b (Table 3). The variability per gene of the tissue/fecal FCoVs of each cat and in the challenge stock virus was small and ranged from 0.051 to 0.553%. While the 3a, 3b, and 3c genes seemed to have equal variability, the 7b gene exhibited a significantly higher mutation frequency than the 3a gene (Figure 2).

**Figure 3:**
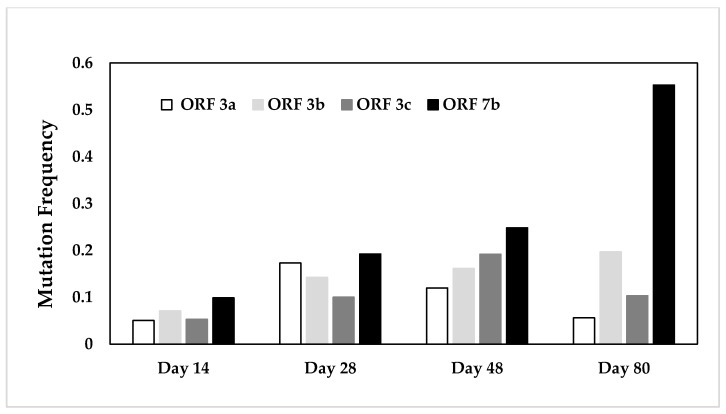
Mean mutation frequencies for ORF3abc and ORF7b in cats grouped according to the timespan between infection and euthanasia. There is a significant difference in the mutation frequency between the different genes over time (p*_KW_* = 0.0463).

We wanted to determine whether the length of infection had an effect on mutation frequencies. The overall comparison of FCoV gene sequences from the different cats euthanized at different time points after infection did not reveal any significant differences (Table 3). Nevertheless, when the cats were grouped according to the timespan passed from infection to euthanasia, a significant difference between the different genes became obvious over time (p*_KW_* = 0.0463). However, the post-test failed to assign the significance to a specific gene, despite a clear increase in the mutation frequency in ORF 7b (Figure 3). SNPs were few and scattered in all genes and all cats. Further, only in 66.2% of the cases did the SNP also result in amino acid changes (Table 3).

#### 2.2.3. Mutations at the Amino Acid Level

Previous studies have provided evidence of a link between truncated 3c proteins and the development of FIP [9,18]. Therefore, we investigated the effect of the mutations and deletions found in the present study on the encoded proteins. Table 4 shows all deletions and SNPs detected in viral sequences from tissue and fecal samples and the challenge stock virus that led to a major alteration, i.e., leading to more than just one amino acid change, of the encoded protein. Deletions were detected in five of the 129 viral gene tissue/fecal sequences. Most mutations of viral sequences led to truncated 7b proteins. Two deletions and one SNP led to truncated 3a and 3c proteins. Two SNPs and one deletion caused the start codon or the stop codon to disappear. The challenge virus only contained one truncated 3b protein.

#### 2.2.4. Phylogenetic Analysis of Sequenced Genes and Identification of a New Recombination Site

**Figure 4:**
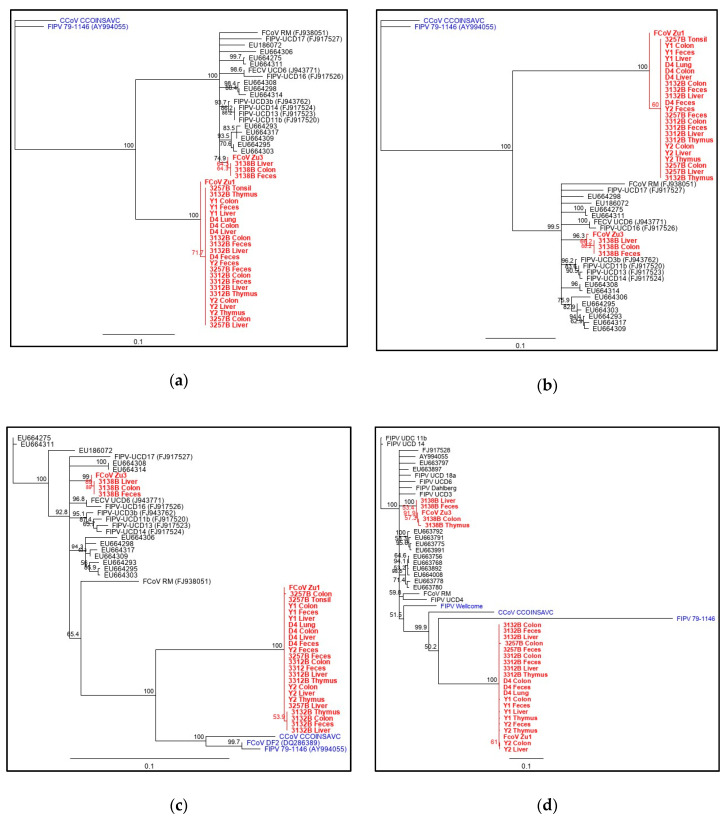
Phylogenetic analysis based on the nucleotide sequences encoding for ORFs 3a (**a**), 3b (**b**), 3c (**c**), and 7b (**d**). The numbers at the nodes were generated from 1000 bootstrap resamplings. The bar represents the mean number of differences per 1000 sites. Bootstrap values below 60 are not depicted. Viral sequences from the present study are in red, serotype II viruses in blue, and serotype I viruses in black.

To determine the serotype of the strains FCoV Zu1 and FCoV Zu3 and their tissue/fecal derivate strains, we constructed bootstrap phylogenetic trees with FCoV and CCoV sequences from the NCBI database (Genbank). Consensus sequences of virus sequences from the same cat and origin and the consensus sequence of the challenge stock virus were aligned to the reference sequences from the Genbank for genes 3a, 3b, 3c, and 7b (Figure 4a–d, respectively). FCoV Zu3 and consensus sequences obtained from cat 3138B inoculated with FCoV Zu3 clustered with type I FCoVs in all genes, whereas in tissue and fecal samples of cats inoculated with the FCoV Zu1 strain, only viral consensus sequences encoding the 3a gene clustered with type I FCoVs. Sequences related to FCoV Zu1 encoding genes 3c and 7b clearly clustered with type II FCoVs. While sequences of the 3c and 7b genes could undoubtedly be assigned to serotype II, because they clustered with the sequences of the corresponding serotype, this was not the case for the 3a and 3b gene. Here, sequences clustered together but could not be unquestionably assigned to a given serotype. Therefore, we speculate that the 3a and/or 3b gene of the challenge virus FCoV Zu1 harbors a new recombination site.

Based on sequence alignments with defined serotype I (FECV-UCD3 (FJ943761)) and serotype II (FIPV 79-1146 (DQ010921)) sequences, the recombination site could be tentatively mapped to the overlapping region between ORF3a and ORF3b (Figure S1).

#### 2.2.5. Evolution Patterns of the Challenge Viruses in Different Cats

To evaluate the sequence evolution patterns of the FCoV Zu1 and Zu3 challenge stock viruses for both the 3abc and 7b genes in each cat (i.e., at different time points post infection), bootstrap phylogenetic trees were constructed, with each available sequence obtained from different colonies in relation to the respective challenge stock virus consensus sequence (Supplementary Figures S2–S5). In cats 3132B, 3138B, 3312B (Figures S2 and S3), and D4 (Figures S4 and S5), the variability between the different sequences was very small and no subtree resulted from the analysis of either gene. The virus variation seemed to be evenly distributed across tissue and fecal samples independent of the time interval between infection and euthanasia (14, 28, or 48 days). In cat Y2, the gene 3abc based analysis revealed an equal distribution of virus variability in all examined tissue and fecal samples (Figure S4). However, for the 7b gene, the analysis revealed a higher variability, with viral sequences from different origin grouping to a subtree. The evolutionary pressure on the 7b gene seemed to be similar in all samples of this cat (Figure S5). Phylogenetic analysis of cat Y1 sequences based on genes 3abc and 7b showed subtrees in both genes. The sequence variability in the 3abc genes was smaller than in the 7b gene (Figures S4 and S5). Analysis based on the 7b gene led to three different subtrees where viral sequences of the same tissue and feces grouped together and were clearly distinct from each other. Also, in cat 3257B, the variability in the 7b gene was slightly higher than in the 3abc genes (Figures S4 and S5). Trees constructed with both genes formed subtrees. Two colonies sequenced from the colon of this cat grouped together when the analysis was based on the 3abc genes. Analysis of the 7b gene yielded two subtrees, one consisting of two sequences from the feces and one of each feces and a colon sequence. The genetic difference of the viral sequences identified in the colon of cat 3257B to FCoV Zu1 (Figures S4 and S5) was the highest of all sequences analyzed, potentially reflecting the longer time span after infection (80 days) in this animal.

#### 2.2.6. Evolution Patterns of the Challenge Virus FCoV Zu1 in the Different Organs

**Figure 5:**
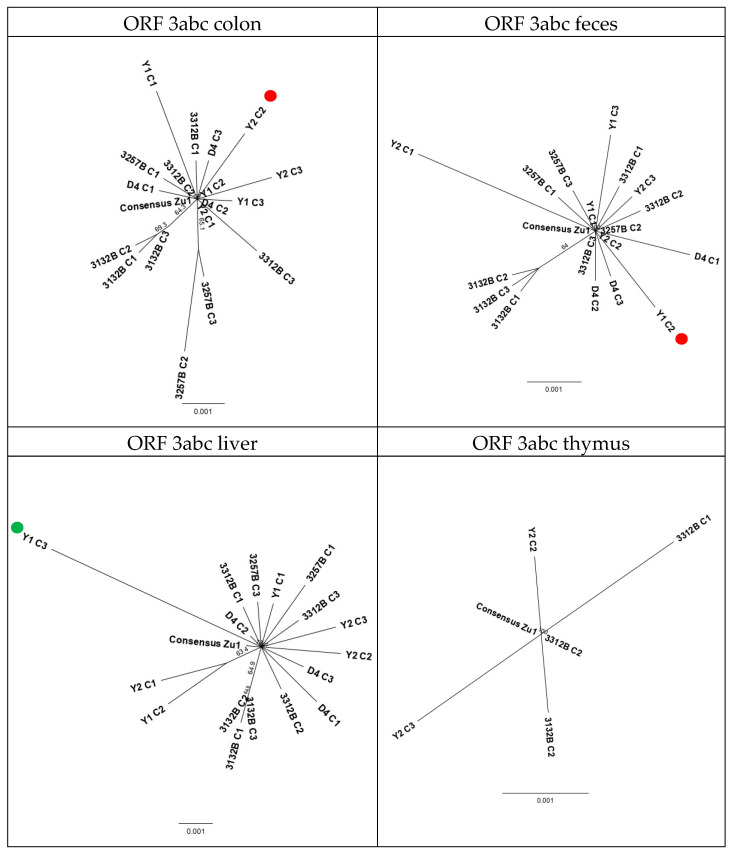
Phylogenetic analysis based on the sequences encoding for nonstructural proteins 3abc of all sequences of colon, feces, liver, or thymus, respectively (red dot, sequence carries a deletion that leads to a premature stop codon (Y1 C2) or a shortened protein without frameshift (Y2 C2); green dot, SNP causes disappearance of a stop codon (Y1 C3); bar, mean number of differences per 1000 sites).

**Figure 6:**
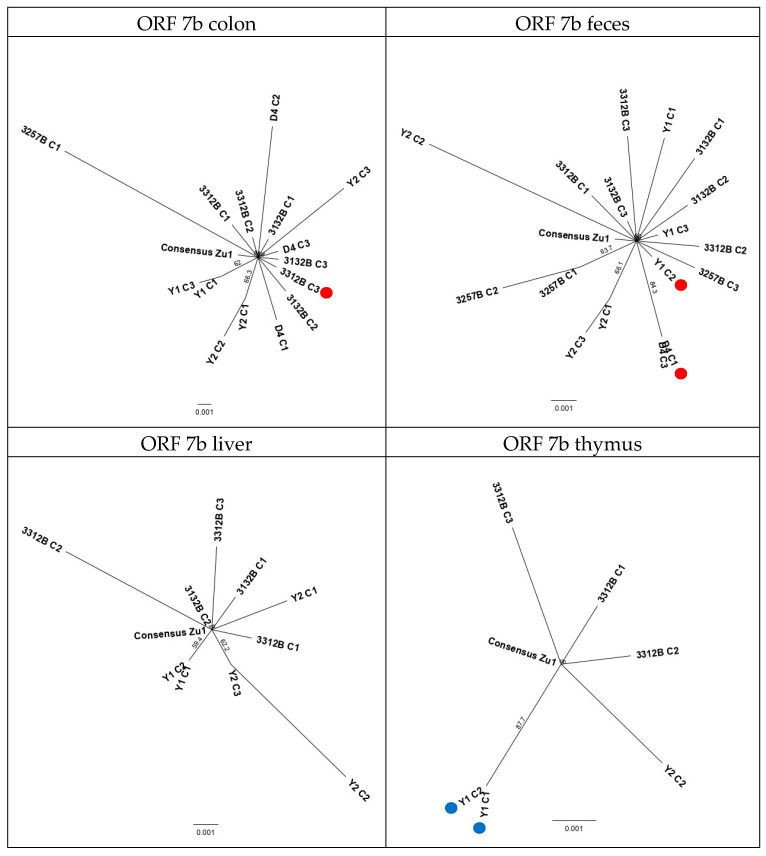
Phylogenetic analysis based on the sequences encoding for the nonstructural protein 7b of all sequences of colon, feces, liver, or thymus, respectively (red dot, sequence carries a deletion that leads to a premature stop codon (3312B C3, Y1C2, D4 C1); blue dot, SNP causes the disappearance of a start codon (Y1 C2, Y1 C1); bar, mean number of differences per 1000 sites).

To assess if the FCoV Zu1 strain evolved divergently in different sites (tissues and/or feces) of individual cats, bootstrap phylogenetic trees were constructed with all sequences from feces and tissues. Figure 5 and Figure 6 show the analysis based on 3abc and 7b genes, with all viral sequences of the same origin. Also, these trees revealed a higher variability in the viral 7b genes (Figure 6) compared to the 3abc genes (Figure 5). With the exception of the tree based on the analysis of the 3abc genes from thymus, all trees generated subtrees (Figure 5: colon; feces; liver). Subtrees consisted of sequences originating from the same cat, except for one subtree for liver sequences where two sequences of cat Y1 and Y2 grouped together (Figure 5: liver). Sequences that diverged in the analysis based on genes 3abc did not originate from the same animals and/or colonies as those diverging in the analysis based on gene 7b. Sequences with mutations that led to truncated proteins did not cluster together but were rather dispersed and the majority were not represented in the subtrees (Figure 5 and Figure 6: red dots). When the analysis was based on genes 3abc of the colon, feces, and liver of cat 3132B, i.e., the cat that was sacrificed after the shortest time post infection (14 days), the sequences always formed a subtree that was clearly distinct from the challenge virus FCoV Zu1 and sequences found in other cats infected with the same virus (Figure 5: colon; feces; liver). This was not seen in the 7b gene analysis (Figure 6: colon; feces; liver). The trees based on the 7b gene indicated that in cats Y1, Y2, D4, and 3257B, the 7b genes were generally more distant from parent FCoV Zu1 than from those of the other cats.

### 2.3. Sequencing of the Spike (S) Gene

**Figure 7:**
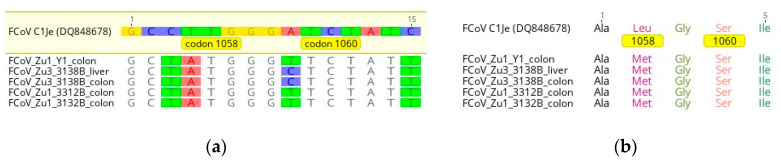
Nucleotide (**a**) and translated amino acid sequences (**b**) of analyzed FCoV strains compared to the FIPV C1Je strain (DQ848678). (**a**) Nucleotides differing from the reference strain at the respective position are colored. Nucleotides coding for codon position 1058 and 1060, respectively, are indicated by yellow boxes (**b**) Positions 1058 and 1060 are indicated by yellow boxes. Ala alanine, Leu leucine, Met methionine, Gly glycine, Ser serine, Ile isoleucine. Images created with Geneious Prime (https://www.geneious.com; Biomatters Ltd., Auckland, New Zealand).

Unfortunately, none of the nested PCR products could be sequenced, neither by direct sequencing nor after cloning. However, sequences were obtained for five of the six samples that were positive after the first RT-PCR step. Four of these sequences derived from the colon and one from the liver. All sequences had an ATG codon at position 1058, resulting in a methionine residue (Figure 7). The FIPV C1Je strain exhibits a TTG codon at this position, resulting in a leucine residue [39]. At position 1060, all sequences had a TCT codon. None of our samples exhibited the serine to alanine mutation described in a minority of FIPVs at this position [33]. Other SNPs detected in the immediate surrounding of position 1058 and position 1060 in our samples did not alter the amino acid composition. Overall, none of the mutations identified by Chang and coauthors in the putative fusion region of the S protein were present [33].

## 3. Discussion

During the last years, many studies have been performed with the aim to identify mutations responsible for the virulence switch in FCoVs towards the FIPV pathotype. There has been evidence that mutations in accessory genes and the S gene of FCoVs are associated with FIP development. So far, most of these studies compared less virulent FCoVs from healthy cats with FCoVs in animals suffering from FIP [18,20,22,40,41,42,43]. In the present study, we investigated the ORFs 3abc and 7b and the S gene mutations of viral sequences identified in different organs and feces of healthy cats that had been experimentally infected with different FCoV field strains and had developed a systemic FCoV infection [14]. In the original study, the challenge virus used for experimental infection had been isolated from the feces of naturally infected cats. Since FCoVs show high mutation rates, due to the infidelity of their RNA-dependent RNA polymerase and homologous recombination events [44], various FCoV variants are transmitted in natural FCoV infections [45]; therefore, due to our experimental setup, we hypothesized a similar scenario. Such virus swarms are called quasispecies, defined by a dominant nucleotide sequence and its mutant spectrum. Evolutionary selection acts on the entire quasispecies rather than on single viral mutants [46]. In our study, we compared the swarm composition of the challenge stock viruses used for infection to the viruses detected in organs and feces at different time points after infection, focusing on the sequences of accessory genes. In parallel, we investigated the S genes for the presence of mutations described to have an impact on the development of FIP [33].

The analysis of the accessory genes of more than 20 colonies of the challenge stock viruses confirmed the existence of one dominant sequence and several mutated derivates in each ORF and strain (data not shown); this observation aligns with the quasispecies theory. In the viral genomes detected in the tissue and fecal samples of the infected cats, the extent of sequences identical to the consensus sequence was lower than in the challenge stock virus. Additionally, viral sequences identified in cats that were euthanized at a later time point after infection overall showed significantly more variation than those from cats that were euthanized earlier, although due to the small number of cats analyzed, this significance could subsequently not be assigned to a specific accessory gene. A limitation for the interpretation of the results is the fact that only few cats (n = 7) were included in this study, of which just one was infected with the FCoV Zu3 strain. Furthermore, two of these animals had been subcutaneously vaccinated with the inactivated FCoV Zu1 strain before challenge with the homologous strain. These cats were included in the study to add a further time point to monitor mutation frequencies over time. As the vaccine was inactivated and unable to replicate, we did not expect to find it in the tissues and feces. The animals seroconverted and started to shed virus in the feces only upon challenge and at the same time, with no differences in titers between vaccinated and non-vaccinated animals. For these reasons, we did not expect to see a difference in mutation frequencies due to vaccination, but we also cannot completely exclude it.

Only three colonies per amplicon of each tissue/fecal sample were sequenced. This sequencing approach was most likely picking the most represented sequences, which might not be enough to appropriately characterize the quasispecies swarm. A targeted high throughput sequencing study would allow for much greater sensitivity for detecting sequence variations in the virus population. Nevertheless, taken together, the results point towards an expansion of the viral swarm with increasing infection time. Yet, stabilization of the virus swarm over a longer period might subsequently occur. This has been shown in a previous study, which also provided evidence that persistently infected cats are likely no source of novel virus variants, since they carry highly conserved FCoV swarms [45].

Single nucleotide polymorphisms (SNP) were found randomly scattered across all accessory genes without defined patterns (hot spots) both in the viral RNA from tissues/feces and the challenge viruses. This suggests that they are a consequence of the infidelity of the viral RNA-dependent RNA polymerase. The present study found a generally higher mutation rate in the 7b gene than in the 3abc genes; the difference was significant for ORF 3a. This confirms previous findings suggesting that the amino acid sequence of the NSP 7b is less conserved than that of other NSPs [27,47]. A bias could have been introduced since a non-proofreading Taq Polymerase was used for PCR amplification to increase the efficiency of the one-step RT-PCR because of the low viral loads in the tissues. But as sequencing errors are supposed to be equally randomly distributed across the genes, this would not impact on the difference of the mutation frequencies. Interestingly, we found deleterious mutations and SNPs that led to truncated NSP 7b proteins in four viral sequences from the feces and colon of different cats infected with the FCoV Zu1 strain. Truncated 7b proteins were previously shown to correlate with attenuated virulence: four well-characterized FCoV strains (FECV 79-1683, FIPV TN406-HP, FIPV UCD2, and FIPV DF2) were found to be avirulent after they had acquired ORF 7b gene deletions during in vitro passage in cell culture. In contrast, field FCoV isolates consistently carried an intact 7b gene regardless of their pathotype [21]. Thus, it was postulated that an intact 7b gene confers a selective advantage in natural infection but is not necessary for in vitro growth [27]. Our study detected truncated 7b proteins solely in either feces or the colon, i.e., the main site of replication and persistence of less virulent FCoVs, possibly representing virus variants that were indeed of low virulence and potentially even unable to infect monocytes/macrophages and to spread systemically. This would support the hypothesis that ORF 7b mutations are not involved in the development of FIP [29]. We also identified each two sequences each with truncated 3a, 3b, and 3c proteins, respectively. These results differ from those of a previous study which sequenced the structural and accessory genes of FCoV from feces and organs of FIP cats and mainly found truncated 3c proteins in the diseased tissue of FIP cats [21]. The same study also claimed that ORFs 3a, 3b, and 7a show the least variability among the other accessory and structural genes. The present study does not support this assumption, but rather indicates that the variability of the ORF 3 genes is equally distributed at least in systemic infections with FCoVs of low virulence. At the same time, the absence of ORF 3c deletions and ORF 7b mutations that have previously been linked to the development of FIP in FCoVs identified in the organs of our cats provides indirect support for the previous hypothesis that these might indeed be a feature of FIPVs [19]. However, since healthy carrier cats also harbor virus in different organs, they are likely not a prerequisite of systemic spread in an infected animal.

The ORFs 3abc and 7b based phylogenetic analysis showed clustering of FCoV Zu3 with serotype I FCoVs, whereas FCoV Zu1 sequences clustered with serotype II FCoVs. This was remarkable since both viruses were identified as type I FCoVs after parts of the S gene had been sequenced in 2004 (DQ256137 and DQ256139) [14]. Serotype definition is commonly based on the reactivity of antibodies to the S protein, hence it is generally accepted that FCoV type I and II can be distinguished based on the S gene sequences [15,48]. The RNA-dependent RNA polymerase of CoVs is well known to be error-prone and to incorporate one mutation per 10 kb [49]; this allows for three mutations per replication cycle of the 30 kb FCoV genome and can lead to deleterious mutations that yield non-viable viruses. To overcome this problem, the viruses might rely on homologous recombination. The latter mostly occurs at specific hotspots in the genome, where secondary RNA structures are formed that cause the polymerase to pause. Four such hotspots, leading to double recombination events that result in type II FCoVs, have previously been identified; upstream ones in the polymerase sequence and downstream ones in genes E and M, respectively [17]. According to this configuration, after a double recombination event, the spike and NSP 3 genes should belong to type II, whereas the NSP 7 should belong to type I. However, the exact locations of these recombination sites vary in the different strains, indicating that serotype II FCoVs continuously arise through independent recombination events [17,50,51]. Therefore, homologous recombination can also generate new variants as long as they do not have evolutionary disadvantages [52]. This might also hold true for FCoV Zu1 where a recombination event in the 3a/3b gene resulted in a genome with type I FCoV S gene, whereas the adjacent 3c gene as well as the 7b gene are those of a type II FCoV.

Controversial evidence exists concerning a set of defined S gene mutations first described in 2012 [33] in FIPVs for which a role in the development of FIP was suggested. Two years later, two studies investigated these mutations in more detail. One study compared fecal samples of healthy FCoV carriers with fecal and/or ascites samples of natural FIP cases by sequencing the accessory and the S genes. This revealed a conserved methionine residue at amino acid position 1,058 in 9/10 FCoVs from healthy carriers, whereas the M1058L mutation was found at this position in 5/6 ascites samples of FIP cats whose feces generally displayed both genotypes. The authors also investigated the animals for ORF 3c mutations/truncations and concluded that these together with mutations at amino acid position 1,058, account for the pathotype switch [22]. Another study, published in the same year, concluded that the spike mutations in question are relevant for systemic spread and are not associated with FIP. The authors found FCoVs that carried the M1058L mutations in the majority of extra-intestinal tissue samples obtained from both cats with FIP and healthy cats, whereas the majority of FCoVs derived from fecal samples had a methionine residue. Percentages of mutated versus conserved sequences in the different types of samples did not significantly differ between FIP cats and controls [35]. We were also interested in the potential presence of these mutations in FCoVs in our cohort of healthy carrier cats. Sequence information covering the region of interest was obtained from four cats and two different organs: colon (four sequences, one per cat) and liver (one sequence). Interestingly, none of these samples exhibited one or both mutations in question (M1058L and S1060A) [33]. These results could indicate that the above-mentioned mutations are not even markers of systemic spread per se [35], but rather of another relevant aspect in the development of FIP that, like systemic spread, is mediated by and/or occurs in monocytes/macrophages. However, our results are based on a single successfully sequenced extra-intestinal sample and are therefore of very limited value. Mutations in the S gene are presumed to be associated with infection of monocytes and macrophages [53], however unfortunately only few samples (2 out of 75) showed a monocyte-associated viremia [14] and due to the RNA degradation, could not be further analyzed. At present, the relevance of the above-mentioned viral mutations for FIP is still not clear. Complementary transparent studies using clearly defined animal populations to obtain sufficient data for a definitive conclusion are clearly lacking and before these are available, no given mutation should be propagated as a diagnostic marker. A real-time RT-PCR has been marketed since August 2014 as a confirmatory diagnostic test for FIP (FIP Virus RealPCR™ Test; IDEXX Reference Laboratories). A detailed description of the investigations on which the test validation was based seems to be lacking; test sensitivity (98.7%) and specificity (100%) were generated in a population of 186 cats “who were either healthy or had confirmed FIP based on biopsy”. It is questionable whether healthy cats are adequate controls for FIP cases. After all, Porter and coworkers found FCoVs with the mutation described by Chang and coworkers [33] not only in 91% of the tissue samples from cats with FIP, but also in 89% of the tissue samples from FCoV positive cats without FIP [35]. Therefore, with the current knowledge, we recommend considering the detection of the S gene mutations in question solely as further support of a diagnosis of FIP, but not as definite proof.

The exact role of the FCoV spike protein in the pathogenesis of FIP is still unknown. It is most likely the key determinant of cell tropism and is crucial for viral host cell entry [32]. Substitution by recombination of the S2 region of FIPV 79-1146 into FECV 79-1683 was found to be sufficient to enable the hybrid virus to effectively infect macrophages in vitro [30]. Thus, it is likely not the receptor binding (via S1) but the fusion process that is critical in this context. Also, for type I FCoVs, which, unlike type II FCoVs, hardly grow in cell culture, the receptor is not yet known. Interestingly, a cell culture adapted type I FCoV strain (UCD 1) was found to carry a mutation in the furin cleavage site that renders the S protein susceptible to cleavage by heparan sulfate [54], leading to the speculation that the amino acid composition of the furin cleavage site is critical for cell culture adaption and thus cell tropism. Indeed, a strong correlation was later found between conservation of the furin cleavage site and FCoV pathotype [36]. Unfortunately, attempts to target the furin cleavage site at the boundary of S1 and S2 [36] did not yield conclusive results in our study (data not shown).

FIP pathogenesis includes several key processes, i.e., the establishment of systemic FCoV infection; effective and sustainable viral replication in monocytes/macrophages; and activation of infected monocytes [6]. Efficient entry into monocytes/macrophages is an essential prerequisite for all these, but there are most likely additional S protein-unrelated factors that allow the increase in viral replication in monocytes/macrophages and their subsequent activation. Therefore, S gene mutations likely only represent contributing factors among multiple events, ultimately resulting in FIP.

In conclusion, despite some limitations mentioned above, using known field viruses and a controlled experimental setting, we were able to establish that FCoVs in the tissue and fecal samples of healthy FCoV infected cats do not carry the ORF 3abc, 7b, and S gene mutations that have previously been linked to the development of FIP. These findings support the hypothesis that these alterations are linked to FIPV pathotype viruses. However, like all other studies so far, they do not answer the question whether their occurrence does directly cause FIP. Interestingly, the viruses detected in the tissues also lacked mutations seen in association with systemic spread of FCoVs [35], which indicates that they are not essential for this key prerequisite of FIP. Furthermore, we detected a homologous recombination site in the strain FCoV Zu1 that has not been described before. Further comparative studies, and in particular, those using a NGS approach, are required to ultimately assess whether the development of FIP can be attributed to virus characteristics alone or whether it represents an interplay between FIPV pathotype viruses and a host prone to a specific ultimately detrimental immune response.

## 4. Materials and Methods

### 4.1. Sample Characteristics

The tissue and fecal samples used in this study originated from a study performed between 2000 and 2003 [14]. The study was approved by the Swiss ethics committee (TVB66-2000). Specific pathogen-free (SPF) cats aged between 8 and 16 weeks were orally infected with different FCoV type I strains (FCoV Zu1 [DQ256137.1] or FCoV Zu3 [DQ256139.1]) isolated from feces of healthy field cats or from the intestines of cats that had been experimentally infected with the same virus strains. Cats D4, Y1, and Y2 originated from an unpublished study where the cats had been subcutaneously vaccinated with the inactivated homologous FCoV strain 10, 7, and 4 weeks before challenge. Cats D4 and Y1 had been vaccinated with the inactivated strain mixed 1:1 with the adjuvant Diluvac Forte (Intervet Ltd., UK). For cat Y1, a CpG motif, as described in a previous vaccine study [55], had been added together with the adjuvant. Cat Y2 had been mock vaccinated. The challenge virus (1 mL) was administered twice, as previously described [14]. All infected cats had remained clinically healthy until euthanasia at 14, 28, 48, or 80 days after infection (Table 1). A full postmortem examination was carried out. The gross and histological examination did not reveal any pathological changes apart from lymphatic hyperplasia [14]. Challenge stock virus samples of the FCoV Zu1 and Zu3 strains originated from the same material originally prepared and aliquoted for the experimental challenge. All samples used in the current study had been stored at −80 °C since 2004.

### 4.2. Viral RNA Isolation from Tissues and Feces

Feces, colon, liver, and thymus or, when the thymus was not FCoV positive by RT-qPCR, tonsils or lung, were analyzed. Viral RNA was isolated with the RNeasy Mini Kit (Qiagen, Hombrechtikon, Switzerland). Approximately 25 mg of tissue was taken from the frozen samples and placed in a 2 mL Eppendorf tube containing 600 μL of Qiagen Lysis Buffer RLT, 3.5 μL β-mercaptoethanol, and a 3 mm steel bead (Schieritz & Hauenstein AG, Arlesheim, Switzerland). Samples were homogenized and lysed by mixing at 30 Hz for 1 min in a Mixer Mill 300 device (Qiagen). After mixing, samples were centrifuged shortly to reduce the foam that had formed during the homogenization step. The homogenate was loaded on a QIAshredder spin-column (Qiagen) and centrifuged at 14,000 rounds per min (RPM) for 2 min; 350 μL of 70% ethanol were added to the flow-through and mixed by pipetting. Samples were transferred to RNeasy spin columns placed in 2 mL collection tubes. Downstream operations were performed according to the manufacturer’s instructions and RNA was eluted with 50 μL nuclease free H_2_O and centrifugation at 10,000 RPM for 1 min.

### 4.3. Quantitative FCoV Real-Time Reverse Transcription PCR (RT-qPCR)

FCoV loads were determined by a RT-qPCR assay that detects a 102 bp long amplicon of the 7b gene, modified from previously described protocols [56]. Briefly, 25 μL reactions contained 12.5 μL 2× Reaction buffer (One step RT qPCR MasterMix Plus Low ROX, RT-QPRT-032XLR, Eurogentec, Seraing, Belgium), 0.375 μL each of forward and reverse primers (20 μM) (Microsynth, Balgach, Switzerland), 0.75 μL probe (Microsynth), 0.125 μL EuroScript Reverse Transcriptase & RNase Inhibitor (Eurogentec), 5 μL template RNA, and RNase free water to 25 μL. The reaction underwent reverse transcription (RT) at 48 °C for 30 min, denaturation for 10 min at 95 °C, and 45 cycles of 95 °C for 15 s and 60 °C for 1 min. To test for any inhibition, each RNA sample was tested neat and after 10-fold dilution in RNase free water. All qPCR assays were performed using an ABI 7500 Fast Sequence Detection System (Applied Biosystems, ThermoFisher Scientific, Reinach, Switzerland). Positive and negative controls were included in each RT-qPCR run.

### 4.4. One-Step RT-PCR and Nested PCR for Specific Gene Targets

Synthesis of cDNA and PCR amplification were performed with the SuperScript III One-Step RT-PCR System with Platinum Taq DNA Polymerase (Invitrogen, Basel, Switzerland) using specific primers. Viral RNA isolated from a cell culture supernatant infected with the FCoV Wellcome strain or from the FCoV Zu1 and FCoV Zu3 gut homogenates used for infection of the cats was always included as positive controls. Also, two negative controls were included, of which one was kept open throughout the manipulation to control for airborne contaminations. PCR reactions (25 μL) contained 12.5 μL 2× Reaction Mix (provided in kit), 1 μL SuperScript III RT/Platinum Taq Mix, 0.5 μL each of forward and reverse primers (10 μM) (Microsynth), 4 μL template RNA, and autoclaved distilled water to 25 μL.

Three novel assays were developed. One amplified parts of the S gene, the 3abc genes, the E gene, and parts of the M gene of the strain FCoV Zu1. Primers used for this amplicon (S-M, 1845 bp) were FCoV_SE_f (5′-TGC TGT TTA ACT ACT GGT TGT TGT GGA-3′) and FCoV_SM_r (5′-GCA CCC GCT ATA CTA AGG CCG-A 3′). The second assay amplified parts of the S gene, the 3abc genes, and parts of the E gene of the strain FCoV Zu3. Primers used for this amplicon (S-E, 1348 bp) were FCoV_SNSP3b.f (5′-CTT GGT ATG TGT GGC TAC TAA TTG G-3′) and FCoV_SE_r (5′-ATC AAC AGG AGC CAG AAG AAG ACA CT-3′). The third assay amplified parts of the 7a and the whole 7b gene of both strains. For this amplicon (NSP7ab, 855 bp), already described primers were used, namely 7a-F1 (5′-CTG CGA GTG ATC TTT CTA G-3′) [29] and FCoV1229r (5′-AAC AAT CAC TAG ATC CAG ACG TTA GCT-3′) [56]. All assays were run on a Biometra TPersonal thermocycler (Labgene, Chatel-St-Denis, Switzerland). Cycling conditions were 55 °C for 30 min, 94 °C for 2 min; 40 cycles of 94 °C for 15 s, 60 °C for 30 s, and 68 °C for 2 min; 5 min at 68 °C and then cooling to 10 °C for the first assay (S-M); and 55 °C for 30 min, 94 °C for 2 min; 40 cycles of 94 °C for 15 s, 55 °C for 30 s, and 68 °C for 1 min; 5 min at 68 °C and then cooling to 10 °C for the second and third assay (S-E and NSP7ab).

For the amplification of the S gene targeting the mutations M1058L and S1060A, a modified previously published nested RT-PCR assay was used [33]. The FCoV-UCD1-S.3022 (5′-CAA TAT TAC AAT GGC ATA ATG G-3′) forward and FCoV-UCD1-S.3636 (5′-CCC TCG AGT CCC GCA GAA ACC ATA CCT A-3′) reverse primer were used for a first amplification (amplicon 615 bp) using the one-step RT-PCR kit (SuperScript III RT/Platinum Taq Mix, Invitrogen). The reaction had the same composition as above. Cycling conditions were 55 °C for 30 min, 94 °C for 2 min; 40 cycles of 94 °C for 15 s, 47 °C for 30 s, and 68 °C for 2 min; 5 min at 68 °C and then cooling to 10 °C. For the second nested PCR step, if needed, two newly designed primer pairs amplifying the region of interest (amplicon 134 bp), FCoV-UCD1-S.3027f (5′-AAT GGT GCT TCC TGG GGT TG-3′) and FCoV-UCD1-S.3160r (5′-GCA CCT GCA TAG CAA AAG GC-3′), and the Phusion Hot Start II High-Fidelity DNA Polymerase (Thermo Scientific Scientific) were used. Then, 5 μL of one-step RT-PCR product were added to 20 μL of Mastermix (composed of 5 μL 5× HF Buffer, 0.5 μL d’NTPs (10 mM), 0.625 μL of each primer (20 μM), 0.5 μL Phusion High-Fidelity Polymerase (2 U/μL), and 12.75 μL RNase-free water). Cycling conditions were 98 °C for 3 min; 40 cycles of 98 °C for 10 s, 59 °C for 30 s, and 72 °C for 2 min; 10 min at 72 °C and then cooling to 10 °C.

### 4.5. Purification of PCR Products

Gel electrophoresis was done with 1.5% agarose gels containing 0.1 mM GelRed™ (Biotium, Hayward, ON, Canada). After the addition of Orange G 6× loading dye (Bioconcept, Allschwil, Switzerland) in a 1:5 ratio to the amplified DNA, samples were loaded on the gels and run at 100 V. A 1-kilobase-pair DNA ladder (Fermentas, St. Leon-Rot, Germany), or alternatively, a Gene Ruler DNA Ladder Mix (Thermo Scientific) or a 10-kilobase-pair DNA Ladder (Eurogentec), was used for molecular size comparisons. Appropriate bands were cut out with sterile razor blades and weighed. PCR bands were purified with the MinElute or QIAquick Gel extraction Kit (Qiagen) according to the manufacturer’s instructions.

### 4.6. Cloning and Sequencing

The PCR amplicons were cloned into the pCR4 plasmid with the TOPO TA Cloning Kit for Sequencing (Invitrogen) using TOP 10 competent cells according to the manufacturer’s instructions. Transformed TOP 10 cells were grown overnight at 37 °C on Luria Broth-Plates containing kanamycin. From each cloned RT-PCR amplicon, three colonies for each tissue/fecal sample as well as 23 and 21 colonies of the FCoV Zu1 and FCoV Zu3 challenge stock virus strains (for better characterization of the virus pool used for infection), respectively, were picked and cultured overnight at 37 °C in Luria-Bertani-Liquid-Medium containing ampicillin. Cultures were centrifuged at 7900 RPM for 3 min and the pellets were used for further manipulation. Plasmids were isolated from the TOP 10 cell pellets with the QIAprep Miniprep Kit (Qiagen) according to the manufacturer’s instructions. Concentrations of the eluted DNA were determined using a Nanodrop 2000c (NanoDrop products, Wilmington, DE, USA). Sanger sequencing was performed by a commercial laboratory (Microsynth) using M13 forward and M13 reverse primers on 800 ng plasmid DNA. The nested PCR amplicons were either sequenced directly using the corresponding primers or cloned into the pCR4 plasmid with the TOPO TA Cloning Kit for Sequencing (Invitrogen), as described above, after adding A-overhangs using the Taq DNA Polymerase (Sigma-Aldrich). The reaction mix for A-tailing contained 5 μL of PCR product, 1.2 μL Taq Buffer (10×), 0.24 μL dATP (10 mM), 0.1 μL Taq DNA Polymerase (5 U/μL), and 5.46 μL RNase-free water and was incubated at 72 °C for 15 min. Sequence accession numbers are as follows: (FCoV Zu1 challenge stock virus ORF3abc: MT606018-MT606040; FCoV Zu1 tissue/fecal virus ORF3abc: MT606041-MT606103; FCoV Zu3 challenge stock virus ORF3abc: MT551809-MT551829; FCoV Zu3 tissue/fecal virus ORF3abc: MT551830-MT551850; FCoV Zu1 challenge stock virus ORF7b: MT551786-MT551808; FCoV Zu1 tissue/fecal virus ORF7b: MT606104-MT606152; FCoV Zu3 challenge stock virus ORF7b: MT551830-MT551850; FCoV Zu3 tissue/fecal virus ORF7b: MT551860-MT551866; FCoV Zu1 & Zu3 tissue virus partial S-gene: MT551867-MT551871).

### 4.7. Phylogenetic Analysis

Phylogenetic and molecular evolutionary analyses were conducted using Geneious Prime (Biomatters Ltd.). Nucleotide sequences were edited, assembled, and aligned. Alignments were manually adjusted when necessary. Bootstrap phylogenetic trees were constructed using the Neighbor-Joining algorithm and the Tamura-Nei genetic distance model [57]. Four bootstrap phylogenetic trees based on the 3a, 3b, 3c, or 7b genes were constructed with the consensus sequence of all viral RNA sequences from the same tissue or feces of each cat and different FIPV, FCoV, and CCoV strains retrieved from the Genbank. Additional Neighbour-Joining trees were constructed with either all 3abc or 7b sequences of the same cat or with all sequences from the same sample. The consensus sequences of FCoV Zu1 and FCoV Zu3 served as outgroups.

### 4.8. Statistics

Variable sites and deletions of genes 3a, 3b, 3c, and 7b were analyzed with Graph Pad Prism Version 8.4.2 (San Diego, CA, USA). Data were tested for normal distribution with the Kolmogorov-Smirnov test. Statistical significance was determined using one-way ANOVA analysis of variance with Bonferroni correction to compare the mutational frequency of the different genes. Mean mutation frequencies in challenge stock viruses and viral sequences from tissues/feces for ORF 3abc and ORF 7b of FCoV Zu1 and Zu3 were evaluated using the Wilcoxon signed-rank test. The time-dependent mutation frequency between genes was analyzed using Kruskal-Wallis with Dunn’s multiple comparison posttest. Data were expressed as median with boxes and whiskers showing the maximum and minimum values within a group or as means in a bar chart. A *p*-value < 0.05 was considered statistically significant in all cases.

## Figures and Tables

**Table 1:** Characteristics of the samples used in this study.

Cat ID	Challenge Virus Strain (Origin)	Timepoint of Euthanasia (Days p.i.)	Virus Load * in Different Organs				Virus Load * in Feces
			Colon	Liver	Thymus	Other	
3132B	FCoV Zu1 (feces)	14	14.9	31.2	40.3		26.4
3138B	FCoV Zu3 (feces)	28	14.2	23.9	42.3		20.5
3312B	FCoV Zu1 (feces)	28	17.0	32.2	37.3		25.3
D4	FCoV Zu1 (gut homogenate)	48	28.4	27.9	negative	39.4 ^a^	28.8
Y1	FCoV Zu1 (gut homogenate)	48	24.2	29.7	35.4		30.3
Y2	FCoV Zu1 (gut homogenate)	48	27.0	38.0	39.0		28.4
3257B	FCoV Zu1 (gut homogenate)	80	27.7	37.9	negative	39.3 ^b^	22.9

p.i. = post infection; * Cycle Threshold (CT) value in RT-qPCR: the lower the CT-value, the higher the viral load; ^a^ Lung; ^b^ Tonsil.

**Table 2:** Number of identical sequences compared to the consensus sequence and single nucleotide polymorphisms (SNPs) in challenge stock virus and feline coronaviruses (FCoVs) sequences obtained from tissues and fecal samples.

Challenge Stock Virus	Gene	Number (Percentage) of Sequences Identical to Consensus ^(1)^		Number (Percentage) of SNPs	
		Challenge Stock	Tissue/Fecal FCoVs	Challenge Stock ^(2)^	Tissue/Fecal FCoVs ^(3)^
FCoV Zu1	ORF	7/23	13/63	28/25668	91/70308
	3abc	(30.4%)	(20.6%)	(0.109%)	(0.129%)
FCoV Zu1	ORF	12/23	7/46	19/14559	75/29118
	7b	(52.2%)	(15.2%)	(0.130%)	(0.256%)
FCoV Zu3	ORF	5/21	2/9	39/22932	16/9828
	3abc	(23.8%)	(22.2%)	(0.170%)	(0.163%)
FCoV Zu3	ORF	9/21	2/8	15/13041	9/4968
	7b	(42.9%)	(25%)	(0.115%)	(0.181%)

^(1)^ Consensus was determined based on all FCoV Zu1 or FCoV Zu3 stock virus sequences. ^(2)^ SNP differences in the stock virus compared to the consensus sequences of FCoV Zu1 or FCoV Zu3 per nucleotides sequenced. ^(3)^ SNP differences in the tissue/fecal viruses compared to the consensus sequences of the FCoV Zu1 or FCoV Zu3 per nucleotides sequenced. ORF: open reading frames.

**Table 3:** Swarm composition: mutations and resulting amino acid changes of the FCoV genes 3a, 3b, 3c, and 7b in tissue and fecal samples of cats at different times post infection (p.i.) and in the challenge stock viruses.

Cat ID/Challenge Virus Stock	Days p.i.	ORF 3a		ORF 3b		ORF 3c		ORF 7b	
		# SNPs/Nts ^(1)^	# AA/SNPs ^(2)^	# SNPs/Nts ^(1)^	# AA/SNPs ^(2)^	# SNPs/Nts ^(1)^	# AA/SNPs ^(2)^	# SNPs/Nts ^(1)^	# AA/ SNPs ^(2)^
3132B	14	1/1980	0/1	2/2820	0/2	4/7530	4/4	5/5064	4/5
		(0.051%)		(0.071%)		(0.053%)		(0.099%)	
3138B	28	4/1917	3/4	5/3213	1/5	9/6426	9/9	7/4347	4/7
		(0.209%)		(0.156%)		(0.140%)		(0.161%)	
3312B	28	3/2178	1/3	4/3102	2/4	5/8283	3/5	17/7596	13/17
		(0.138%)		(0.129%)		(0.060%)		(0.224%)	
D4	48	–	–	4/3102	0/4	13/8283	11/13	11/4431	6/11
				(0.129%)		(0.157%)		(0.248%)	
Y1	48	1/1782	0/1	7/2538	2/7	14/6777	9/14	11/5697	11/11
		(0.056%)		(0.276%)		(0.207%)		(0.193%)	
Y2	48	4/2178	0/4	5/3102	2/5	13/6777	3/13	27/6330	24/27
		(0.184%)		(0.161%)		(0.192%)		(0.427%)	
3257B	80	1/1782	1/1	5/2538	3/5	7/6777	7/7	14/2532	11/14
		(0.056%)		(0.197%)		(0.103%)		(0.553%)	
FCoV Zu1	n.a.	4/4554	2/4	11/6486	7/11	19/17319	13/19	19/14559	14/19
		(0.088%)		(0.170%)		(0.110%)		(0.130%)	
FCoV Zu3	n.a.	9/4899	6/9	15/8211	11/15	23/16422	8/23	15/13041	9/15
		(0.184%)		(0.183%)		(0.140%)		(0.115%)	

Mutation frequencies were calculated over the sum of nucleotides sequenced from all tissue and fecal samples and all colonies. Deletions were counted as one mutation regardless of their length. #: number; Nts: nucleotides; –: no sequence available; p.i.: post infection; n.a.: not applicable. ^(1)^ SNP differences between the tissue/fecal viruses and the consensus sequences of FCoV Zu1 or FCoV Zu3 per nucleotides sequenced. ^(2)^ Number of amino acid changes per SNPs.

**Table 4:** Deletions and SNPs in viral RNA from tissues/feces and stock virus that led to an alteration of the encoded protein.

Cat/Virus	Origin	ORF	SNP or Deletion (# Nts)	Effect on Protein
Y1	Feces	3a	Deletion (1)	Truncation
	Feces	7b	Deletion (1)	Truncation
	Thymus	7b	SNP	No start codon
	Liver	3c	SNP	No stop codon
Y2	Colon	3c	Deletion (3)	Shortening
3312B	Colon	7b	Deletion (4)	Truncation
D4	Feces	7b	Deletion (35)	Truncation
3138B	Liver	3a	SNP	Truncation
FCoV Zu1 challenge stock virus	Gut homogenate	3a	Deletion (22)	No stop codon
		3b	Deletion (22)	Truncation

#: number; Nts: nucleotides.

## Supplementary Materials

The following are available online at https://www.mdpi.com/2076-0817/9/8/603/s1, Figure S1: Nucleotide alignment of the ORF3abc region between FCoV Zu1 and defined Serotype 1 FECV-UCD3 (FJ943761)) and serotype II (FIPV 79-1146 (DQ010921)) viral sequences. Sequences were aligned using the Geneious Prime software (www.geneious.com, Biomatters Ltd.) and the Muscle method. Dots depict identical nucleotides. The grey bars indicating the different ORF3abc locations are based on the sequence of FCoV Zu1. Figure S2: Phylogenetic analysis based on the sequences encoding for nonstructural proteins 3abc for cats 3132B, 3138B, and 3312B. (*, day p.i. of euthanasia; yellow dot, sequence carries SNP that leads to a premature stop codon; bar, mean number of differences per 1000 sites), Figure S3: Phylogenetic analysis based on the sequences encoding for nonstructural protein 7b for cats 3132B, 3138B, and 3312B. (*, day p.i. of euthanasia; red dot, sequence carries a deletion that leads to a premature stop codon; bar, mean number of differences per 1000 sites). Figure S4: Phylogenetic analysis based on the sequences encoding for nonstructural proteins 3abc for cats D4, Y1, Y2, and 3257B. (*, day p.i. of euthanasia; red dot, sequence carries a deletion that leads to a premature stop codon (Y1) or a shortened protein without frameshift (Y2); green dot, SNP causes disappearance of a stop codon; bar, mean number of differences per 1000 sites). Figure S5: Phylogenetic analysis based on the sequences encoding for nonstructural protein 7b for cats D4, Y1, Y2, and 3257B. (*, day p.i. of euthanasia; red dot, sequence carries a deletion that leads to a premature stop codon; blue dot, SNP causes disappearance of a start codon; bar, mean number of differences per 1000 sites).
