# Supplementary material for: FCoV Viral Sequences of Systemically Infected Healthy Cats Lack Gene Mutations Previously Linked to the Development of FIP
Source: Pathogens. 2020 Jul 24;9(8):603. doi: 10.3390/pathogens9080603 (PMC7459962; doi:10.3390/pathogens9080603)
Supplement: Supplementary file 1 [file pathogens-09-00603-s001.zip › pathogens-837380-supplementary materials/Supplementary Figures S2 new.pdf]

<table>
<tr><th>*</th><th>FCoV Zu1 from feces</th><th>FCoV Zu3 from feces</th></tr>
<tr><td>14</td><td>3132B ORF 3abc
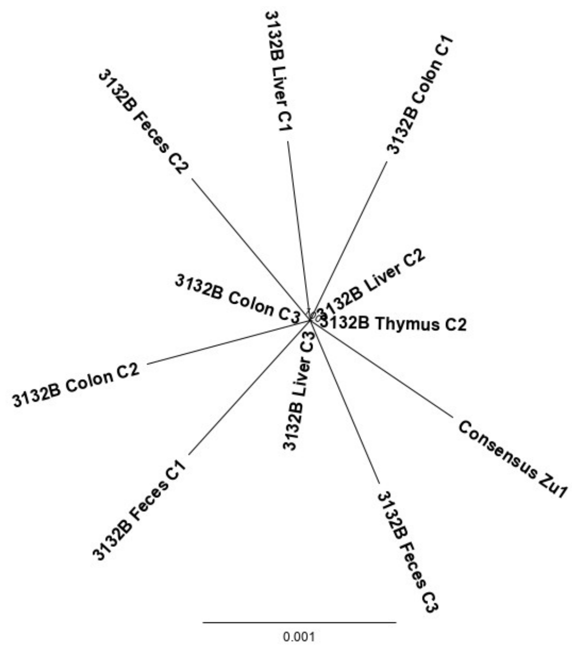
</td><td></td></tr>
<tr><td>28</td><td>3312B ORF 3abc
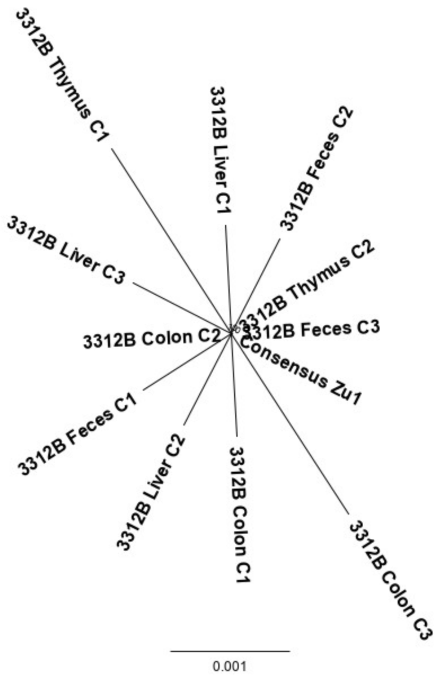
</td><td>3138B ORF 3abc
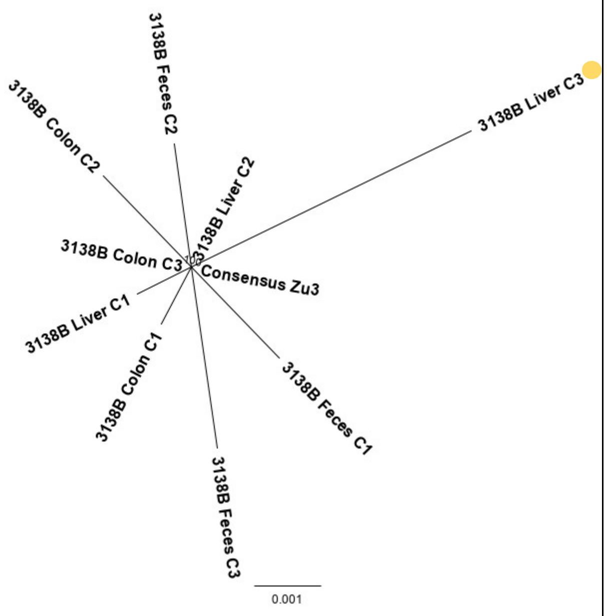
</td></tr>
</table>

Figure S2. Phylogenetic analysis based on the sequences encoding for nonstructural proteins 3abc for cats 3132B, 3138B, and 3312B. (*, day p.i. of euthanasia; yellow dot, sequence carries SNP that leads to a premature stop codon; bar, mean number of differences per 1000 sites).
