# Supplementary material for: FCoV Viral Sequences of Systemically Infected Healthy Cats Lack Gene Mutations Previously Linked to the Development of FIP
Source: Pathogens. 2020 Jul 24;9(8):603. doi: 10.3390/pathogens9080603 (PMC7459962; doi:10.3390/pathogens9080603)
Supplement: Supplementary file 1 [file pathogens-09-00603-s001.zip › pathogens-837380-supplementary materials/Supplementary Figures S4 new.pdf]

<table>
<tr><td>*</td><td colspan="2">FCoV Zu1 from gut homogenate</td></tr>
<tr><td rowspan="2">48</td><td>D4 ORF 3abc</td><td>Y1 ORF 3abc</td></tr>
<tr><td>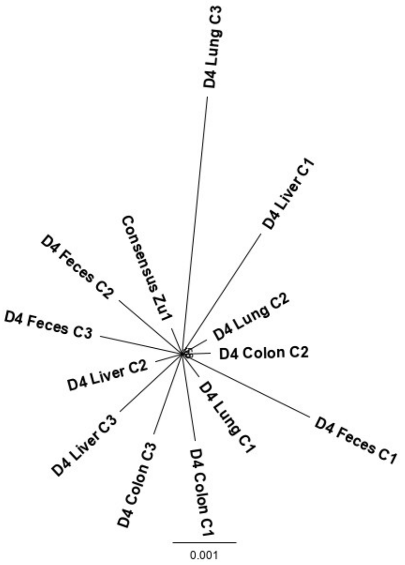
</td><td>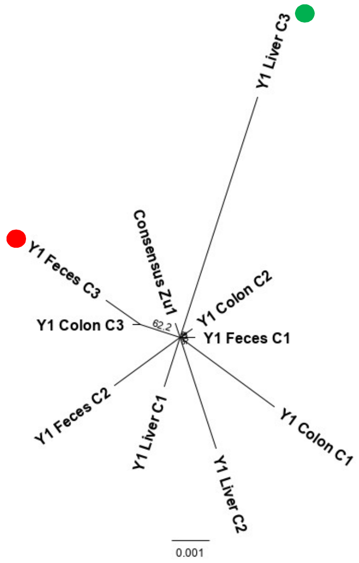
</td></tr>
<tr><td rowspan="2">48</td><td>Y2 ORF 3abc</td><td></td></tr>
<tr><td>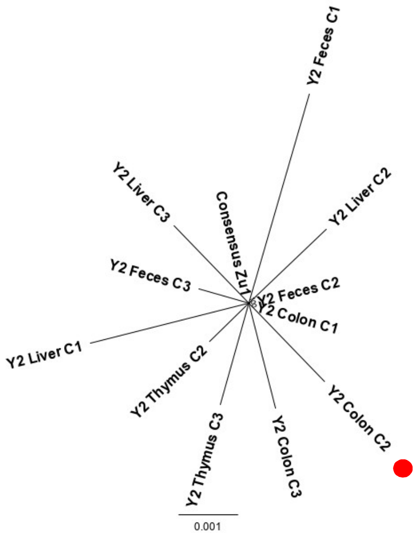
</td><td></td></tr>
<tr><td rowspan="2">80</td><td>3257B ORF 3abc</td><td></td></tr>
<tr><td>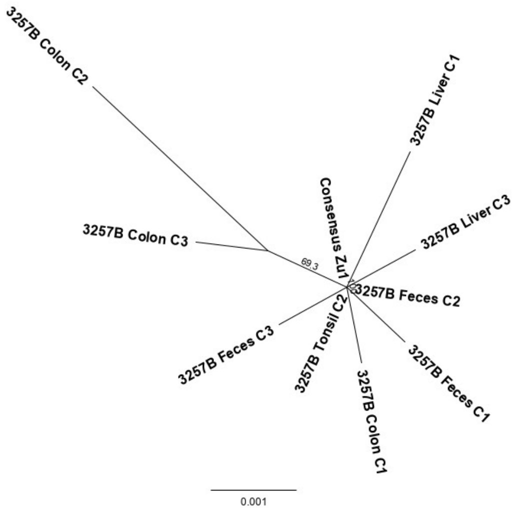
</td><td></td></tr>
</table>

Figure S4. Phylogenetic analysis based on the sequences encoding for nonstructural proteins 3abc for cats D4, Y1, Y2, and 3257B. (*, day p.i. of euthanasia; red dot, sequence carries a deletion that leads to a premature stop codon (Y1) or a shortened protein without frameshift (Y2); green dot, SNP causes disappearance of a stop codon; bar, mean number of differences per 1000 sites).
