# Supplementary figures and images for: FCoV Viral Sequences of Systemically Infected Healthy Cats Lack Gene Mutations Previously Linked to the Development of FIP
Source: Pathogens. 2020 Jul 24;9(8):603. doi: 10.3390/pathogens9080603 (PMC7459962; doi:10.3390/pathogens9080603)

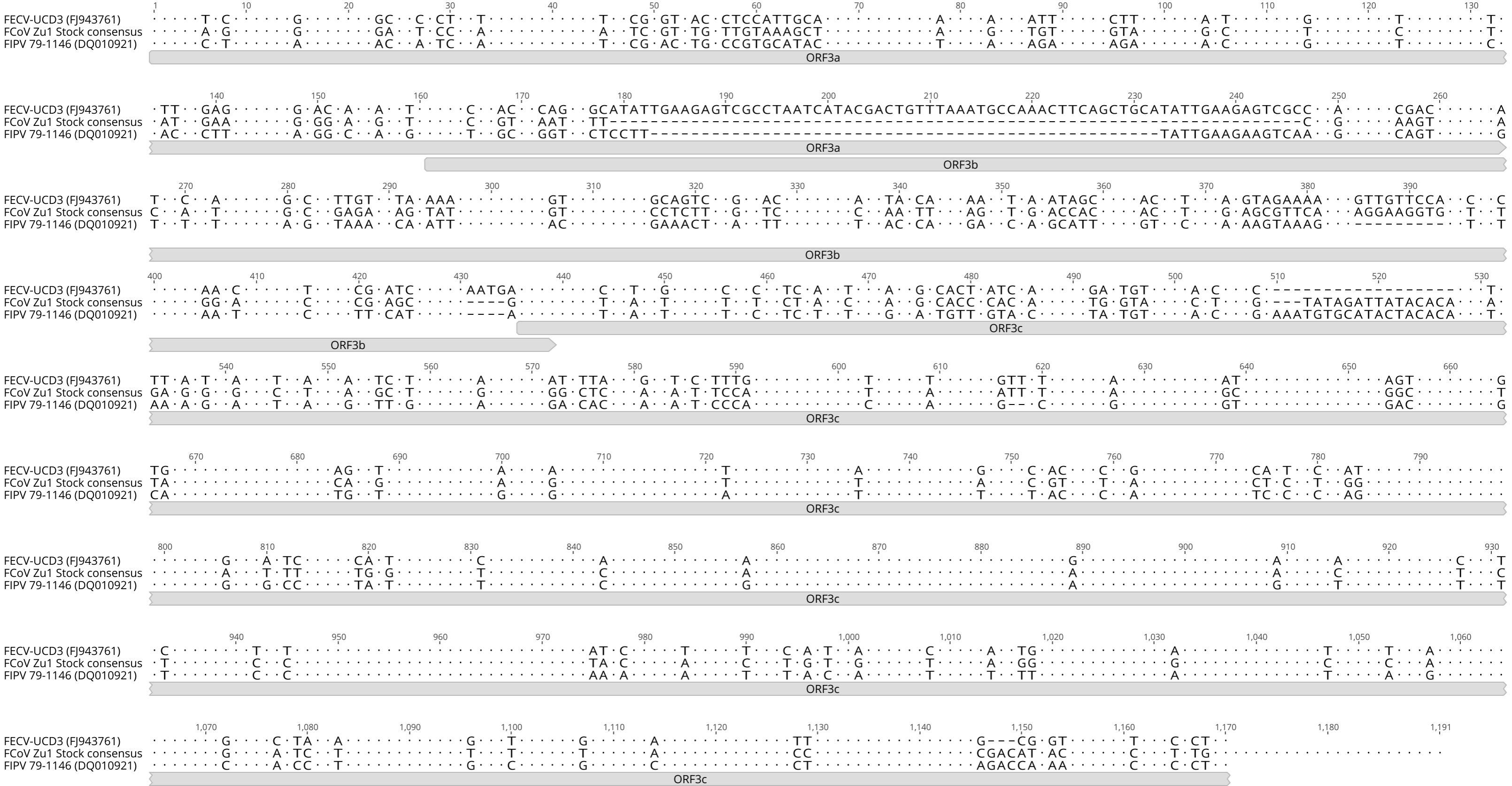

Supplement: Supplementary file 1 [file pathogens-09-00603-s001.zip › pathogens-837380-supplementary materials/Supplementary Figure S1.pdf]
